# Natural killer cell subsets and their functional molecules in peripheral blood of the patients with breast cancer

**DOI:** 10.1002/iid3.1255

**Published:** 2024-04-23

**Authors:** Reza Darvishvand, Somayeh Rezaeifard, Razie Kiani, Sedigheh Tahmasebi, Zahra Faghih, Nasrollah Erfani

**Affiliations:** ^1^ Department of Immunology, School of Medicine Shiraz University of Medical Sciences Shiraz Iran; ^2^ School of Medicine, Shiraz Institute for Cancer Research Shiraz University of Medical Sciences Shiraz Iran; ^3^ Breast Diseases Research Center Shiraz University of Medical Sciences Shiraz Iran

**Keywords:** breast cancer, natural killer (NK) cells, NK cell functional molecules, NK cell subsets

## Abstract

**Background:**

Natural killer (NK) cells, CD3^−^ lymphocytes, are critical players in cancer immune surveillance. This study aimed to assess two types of CD3^−^ NK cell classifications (subsets), that is, convectional subsets (based on CD56 and CD16 expression) and new subsets (based on CD56, CD27, and CD11b expression), and their functional molecules in the peripheral blood of patients with breast cancer (BC) in comparison with healthy donors (HDs).

**Methods:**

Thirty untreated females with BC and 20 age‐matched healthy women were enrolled. Peripheral blood samples were collected and directly incubated with fluorochrome‐conjugated antibodies against CD3, CD56, CD16, CD27, CD11b, CD96, NKG2C, NKG2D, NKp44, CXCR3, perforin, and granzyme B. Red blood cells were then lysed using lysing solution, and the stained cells were acquired on four‐color flow cytometer.

**Result:**

Our results indicated 15% of lymphocytes in peripheral blood of patients with BC and HDs had NK cells phenotype. However, the frequency of total NK cells (CD3^−^CD56^+^), and NK subsets (based on conventional and new classifications) was not significantly different between patients and HDs. We observed mean fluorescent intensity (MFI) of CXCR3 in total NK cells (*p* = .02) and the conventional cytotoxic (CD3^−^CD56^dim^ CD16^+^) NK cells (*p* = .03) were significantly elevated in the patients with BC compared to HDs. Despite this, the MFI of granzyme B expression in conventional regulatory (CD3^−^CD56^bright^CD16^−^^/+^) NK cells and CD3^−^CD56^−^CD16^+^ NK cells (*p* = .03 and *p* = .004, respectively) in the patients was lower than healthy subjects.

**Conclusion:**

The higher expression of chemokine receptor CXCR3 on total NK cells in patients with BC may be associated with increased chemotaxis‐related NK cell infiltration. However, lower expression of granzyme B in conventional regulatory NK cells and CD3^−^CD56^−^CD16^+^ NK cells in the patients compared to HDs suggests reduced cytotoxic activity of the NK cells in BC. These results might demonstrate accumulating NK subsets with a dysfunctional phenotype in the peripheral blood of patients with BC.

## Graphical abstract

We observed that natural killer cells in breast cancer have a higher potency to be recruited to the tumor site, but these cells, or at least some of their subsets, may not be able to render their cytotoxic effects.

## 1 INTRODUCTION

Breast cancer (BC) is the most common diagnosed cancer and the main cause of women's cancer death worldwide.^1^, ^2^, ^3^ It has been reported as the 5th leading cause of cancer death in Iranian women, as well.^4^ The role of immune system in breast tumor biology has been well established.^5^, ^6^ In a complex tumor microenvironment (TME), immune cells can either stimulate or inhibit tumor growth, and the final outcome depends on the equilibrium between these two activities.^7^ Natural killer (NK) cells are innate immune effector cells playing vital roles in cancer immune surveillance. These cells are conventionally divided into various subsets including regulatory (CD3^−^CD56^bright^CD16^−^^/+^) and cytotoxic (CD3^−^CD56^dim^CD16^+^) subsets.^8^ NK subtypes have diverse functions in the tumor tissue depending on the functional molecules and activating status.^9^, ^10^ Besides, based on the conventional classification, NK cells with CD3^−^CD56^−^CD16^+^ and CD3^−^CD56^dim^CD16^−^ phenotypes are considered as the exhausted and immature NK cells, respectively. These two phenotypes have been reported to be increased in the peripheral blood of patients with advanced invasive BC.^11^ Alternatively, according to the expression of CD56, CD27, and CD11b, NK cells have been recently categorized into NK^tolerant^ (CD3^−^CD56^bright^CD27^−^CD11b^−^), NK^cytotoxic^ (CD3^−^CD56^dim^CD11b^+^CD27^−^), and NK^regulatory^ (CD3^−^CD56^bright^CD27^+^CD11b^+/^^−^) subsets.^12^

No matter what classification is considered, several functional molecules, including activating, inhibitory, and homing receptors, are expressed on NK cells and are determinant in their activity and/or homing capabilities. Following studies by our research group on the investigation of NK cell subsets and their functional molecules in the tumor tissues ^13^ and tumor‐draining lymph nodes (TDLNs) ^14^ of the patients with BC, in the current study, we aimed to investigate the frequencies of NK subsets, based on both conventional and new classification strategies, as well as the expression of NK cell functional molecules in the peripheral blood of patients with BC and healthy donors (HDs). The latter included functional molecules NKG2D, NKG2C, CD96, and NKP44, chemokine receptor CXCR3, and NK cell intracellular cytotoxic molecules perforin and granzyme B. The association study was then performed between the frequencies of NK subsets and their functional molecules with the patients' clinicopathological characteristics.

## 2 SUBJECTS AND METHODS

### 2.1 Subjects

Just before the mastectomy (or segmental mastectomy), 2 mm of fresh EDTA‐treated peripheral blood samples were collected from 30 women (mean age of 46.53 ± 7.31 years old) with BC referred to Faghihi Hospital, Shiraz, Iran. Clinical and pathological features of the patients are demonstrated in Table 1. The majority of the enrolled patients suffered from invasive ductal carcinoma (IDC, 26/30, 87%), 55% were diagnosed at stage II, and 44.40% were revealed to have resected tumor‐free lymph nodes at the time of surgery. The enrolled patients with BC received no treatments (chemotherapy and radiotherapy) before and/or at the time of sampling. In addition, peripheral blood samples were also obtained from 20 age and sex‐matched healthy (Control) women (mean age of 45.81 ± 4.36 years old) referred to the Medical laboratory of Faghihi Hospital, Shiraz, Iran. Controls were confirmed not to have a history of cancer, autoimmune diseases, and viral or bacterial infections during the last few months before sampling. Informed consent was obtained from all enrolled subjects, and this study was approved by the ethics committee at Shiraz University of Medical Sciences (IR.SUMS.REC.1398.1094).

**Table 1:** Clinical and pathological characteristics of patients with breast cancer subjected to natural killer (NK) cell phenotyping in peripheral blood.

Characteristics	Value (Valid percent)
Age (years ± SEM)	46.53 ± 7.31
Lymph node status	
Free from tumor cells (N0)	12 (44.40%)
Involved by tumor cells	15 (55.60%)
N1 (1–3)	11 (40.70%)
N2 (4–9)	3 (11.10%)
N3 (>9)	1 (3.70%)
Unreported	3
Stage	
I	8 (29.60%)
II	15 (55.60%)
III	4 (14.80%)
IV	0 (0%)
Unreported	3
Tumor type	
Invasive ductal carcinoma (IDC)	26 (87%)
Other types: Ductal carcinoma in situ (DCIC) mucinous carcinoma (MC)	4 (13%)
Tumor size	
T1 (≤2 cm)	11 (42.30%)
T2 (2–5 cm)	14 (53.80%)
T3 (>5 cm)	1 (3.80%)
Unreported	4
Histological grade	
Well differentiated (I)	7 (25%)
Moderately differentiated (II)	16 (57.10%)
Poorly differentiated (III)	5 (17.90%)
Unreported	2

### 2.2 Antibodies and reagents

Fluorescent‐conjugated anti‐human antibodies including fluorescein isothiocyanate (FITC)‐conjugated CD16 (clone: B73.1), phycoerythrin (PE)‐conjugated CD56 (5.1H11), Peridinin‐chlorophyll‐protein complex (PerCP)‐conjugated NKp44 (clone: P44‐8), Allophycocyanin (APC)‐conjugated CD3 (clone: OKT3), FITC‐conjugated CD3 (clone: OKT3), PerCP‐conjugated CD3 (clone: HIT3a), PerCp‐Cy5 conjugated CXCR3 (clone: G025H7), PerCp‐Cy5‐conjugated NKG2D (clone: 1D11), APC‐conjugated CD11b (clone: ICRF44), PerCP‐Cy5.5–conjugated CD27 (clone: M‐T271), PerCP‐Cy5.5‐conjugated perforin (clone: B‐D48), Alexa Fluor® 647‐conjugated granzyme B (clone: GB11), PerCP‐Cy5.5‐conjugated CD96 (clone: NK92.39) and their cognate isotype controls (all from Biolegend) as well as PerCP‐Cy5.5‐conjugated NKG2C (clone: 134591, R&D system) antibodies were used for immunophenotyping. Lysing solution (BD Biosciences) was used for removing the red blood cells. For washing, fixing, and cell permeabilizing, 1X phosphate buffer saline (PBS), 1% paraformaldehyde solution (Sigma), and 1X perm/wash solution (BD Biosciences) were used, respectively.

### 2.3 Surface and intracellular staining

For cellular surface staining, antibodies against CD3, CD16, CD56, CD27, CD11b, NKG2D, NKP44, NKG2C, CD96, and CXCR3 markers, and their cognate isotype controls were directly added to an appropriate amount of blood samples. In the context of the conventional classification of NK cells, fluorescent‐labeled antibodies against CD3, CD56, and CD16 were added into five tubes. After that, an antibody against each functional molecule (CD96, NKG2D, NKG2C, CXCR3, and NKp44) was added to one of the tubes. In addition, for the new classification of NK cells, a sample tube was stained with fluorescent‐conjugated antibodies against CD3, CD56, CD27, and CD11b. The tubes then were incubated for 30 min at room temperature in the dark. To remove red blood cells (RBC), 2 mL of 1X lysing solution was added to each tube and incubated for 10 min at room temperature in the dark. The cells were then centrifuged (300 g, 5 min) and washed twice with 2 mL 1X PBS (200 g, 5 min).

For intracellular staining (ICS) against granzyme B and perforin, the surface‐stained cells were fixed using fixative buffer (15 min at 4°C) followed by permeabilization with 1X Perm/Wash solution (15 min at 4°C). Antigranzyme B and antiperforin or their corresponding isotype control antibodies were then added to the tubes and incubated at 4°C for 30 min. The cells were then washed twice with 1 mL Perm/Wash solution and suspended in 400 µL 1X PBS. The stained samples were acquired on a four‐color flow cytometer (BD FACSCalibur, Biosciences), and the data were subsequently analyzed by FlowJo software package (V10).

### 2.4 Gating strategies for NK cell phenotyping

**Figure 1:**
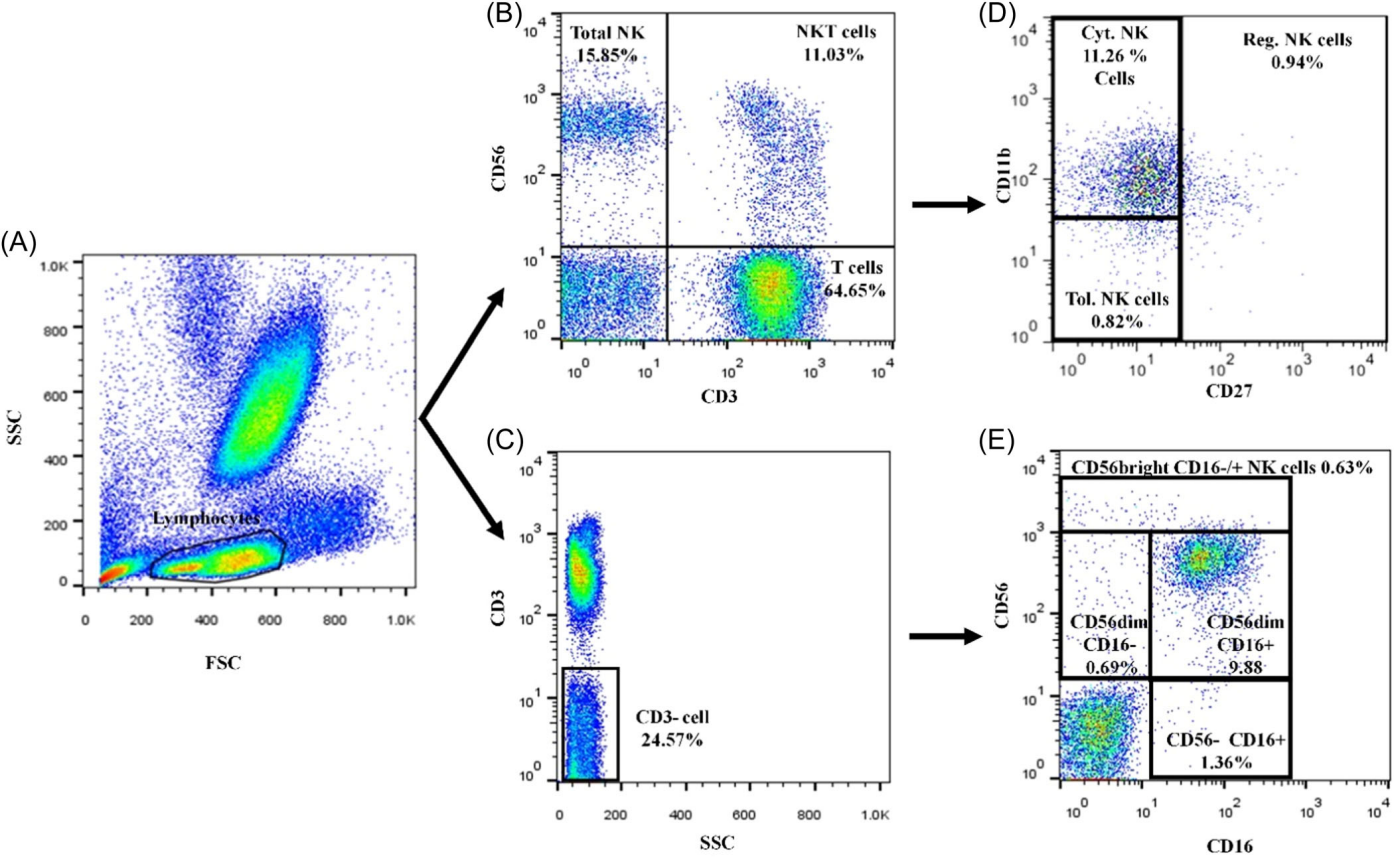
Natural killer (NK) cells subsets in peripheral blood of patients with breast cancer. (A) Lymphocytes gate. (B) NK, NKT, and T cells in lymphocytes gate according to CD3 and CD56 expression. (C) CD3 negative cells in lymphocyte population (D) New classification of NK cells: Total NK cells (CD3^−^CD56^+^) gate was applied on D plot to see different NK cell subsets based on CD11b and CD27 expression, at the top (left) CD11b^+^CD27^−^ (Cyt: cytotoxic NK cells), at the bottom (left) CD11b^−^CD27^−^ (Tol: tolerant NK cells), in the right CD27^+^CD11b^−^^/+^ (Reg: regulatory NK cells). (E) Conventional classification of NK cells: CD3 negative gate was applied on E plot to see different NK cells subsets according to CD16 and CD56 expression, at the top CD56^bright^CD16^−/+^ (Regulatory NK cells), in the middle (left) CD56^dim^ CD16^−^ (Immature NK cells), in the middle (right) CD56^dim^CD16^+^ (Cytotoxic NK cells), at the bottom CD56^‐^CD16^+^ (Exhausted NK cells). All percentages were reported in lymphocyte population.

Gating strategies are shown in Figure 1. As illustrated, total NK (CD3^−^CD56^+^), NKT (CD3^+^CD56^+^), and T cells (CD3^+^CD56^−^) were first gated in lymphocyte population (Figure 1B). To analyze different NK subsets based on CD11b and CD27 expression (New classification), total NK cell gate (CD3^−^CD56^+^) was applied on the plot 1‐D with CD27 in *X*‐axis and CD11b in *Y*‐axis, and the mean (± SEM) percentages of cytotoxic NK cells, tolerant NK cells and regulatory NK cells were shown in the lymphocyte population (Figure 1).

For conventional classification, CD3 negative lymphocyte gate (Figure 1C) was then applied on the plot 1‐E with CD16 in *X*‐axis and CD56 in *Y*‐axis; the mean (±SEM) percentages of different NK cell subsets were shown according to CD16 and CD56 expression in the lymphocyte population (Figure 1).

### 2.5 Statistical analysis

The nonparametric Mann–Whitney *U* and Kruskal–Wallis *H* tests were used to statistically determine differences between various groups, and Dunn's test was applied for *p*‐value adjustment in multiple comparisons. The correlation analysis (between parameters and the frequency of subsets) was evaluated by Spearman correlation test. All data were analyzed by SPSS 22 software package (SPSS GmbH Software), and *p*‐values less than .05 (two‐tailed) was considered significant. Normalized mean fluorescent intensity (MFI) was applied on flow cytometric data to measure the expression of markers. Graphs were drawn by GraphPad Prism 5 software (Inc).

## 3 RESULTS

### 3.1 NK cell subsets in peripheral blood of patients with BC and HDs

**Figure 2:**
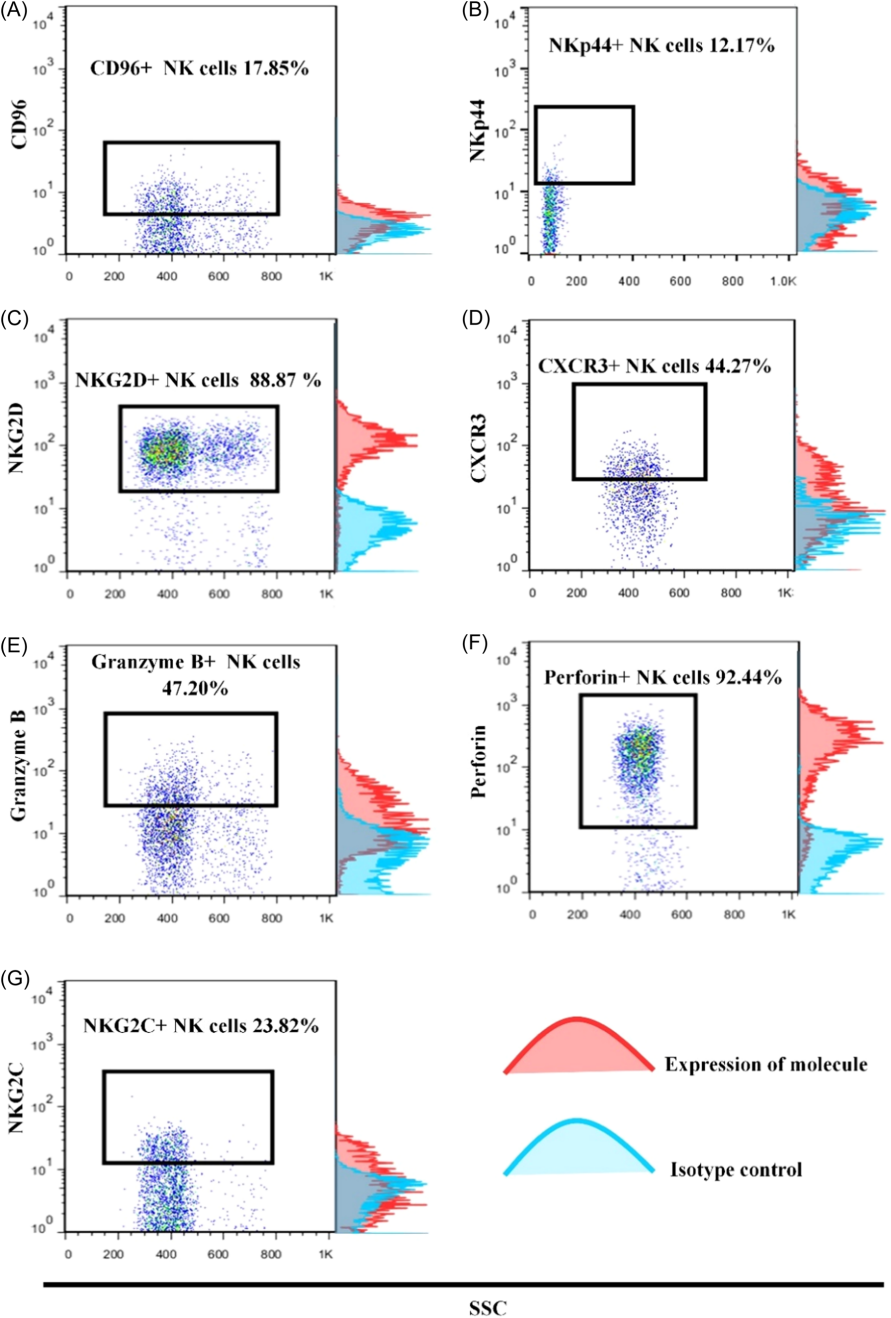
Expression of natural killer (NK) cell functional molecules in patients with breast cancer. Total NK cell gate (CD3^−^CD56^+^, as shown in Figure 1B) was applied on the plots representing functional molecules in one axis versus side scatter (SSC). Mean percentages are reported in total NK cell population (A–G). To ensure more accurate distinction between positive and negative populations, the fluorescence minus one (FMO) was also performed for some molecules (CD96 NKp44, and NKG2C, not shown).

The mean (±SEM) frequencies of NK cell subsets in peripheral blood of the patients with BC and HDs are presented in Table 2. The percentage of total NK cells per lymphocyte population was observed to be 15.85 ± 3.03 and 15.18 ± 1.56 in patients with BC and control subjects, respectively. The difference was not statistically significant (*p* = .32). Comparing frequencies of different NK cell subsets, the results revealed a borderline significance in the lower percentage of conventional cytotoxic NK cells with CD3^−^CD56^dim^CD16^+^ phenotype (9.88 ± 1.30 vs. 12.44 ± 1.49, *p* = .06) and a higher percentage of tolerant NK cells with CD3^−^CD56^+^CD27^−^CD11b^−^ phenotype (0.82 ± 0.13, 0.48 ± 0.10, *p* = .06) in peripheral blood of the patients compared to the HDs. For evaluation of NK cell functional molecules, total NK cell gate (CD3^−^CD56^+^) was applied and the percentages were reported in total NK cells population (Figure 2A–G).

**Table 2:** Mean percentage of natural killer (NK) cells and their subsets among lymphocytes in peripheral blood of the patients with breast cancer (*p*) and healthy donors (HDs).

Cell Subsets	CD Markers	Mean ± SEM *p*	*p*‐Value^a^
		Mean ± SEM HD	
Total NK cells	CD3^−^CD56^+^	15.85 ± 3.03	*p* = .32
		15.18 ± 1.56	
Conventional classification			
Regulatory NK cells	CD3^−^CD56^bright^CD16^−/+^	0.63 ± 0.10	*p* = .31
		0.63 ± 0.08	
CD56^dim^CD16^‐^ NK cells	CD3^−^CD56^dim^CD16^−^	0.69 ± 0.8	*p* = .07
		1.01 ± 0.11	
Cytotoxic NK cells	CD3^−^CD56^dim^ CD16^+^	9.88 ± 1.30	*p* = .06
		12.44 ± 1.49	
CD56^−^ NK cells	CD3^−^CD56^−^CD16^+^	1.36 ± 0.17	*p* = .24
		1.62 ± 0.20	
New classification			
Regulatory NK cells	CD3^−^CD56^+^CD27^+^ CD11b^+/−^	0.94 ± 0.08	*p* = .51
		0.99 ± 0.17	
Cytotoxic NK cells	CD3^−^CD56^+^CD27^−^CD11b^+^	11.26 ± 1.15	*p* = .37
		9.73 ± 1.23	
Tolerant NK cells	CD3^−^CD56^+^CD27^−^CD11b^−^	0.82 ± 0.13	*p* = .06
		0.48 ± 0.10	

^a^ Data are analyzed by the Mann–Whitney *U*‐test.

### 3.2 NK cell subset receptors and effector molecules

**Figure 3:**
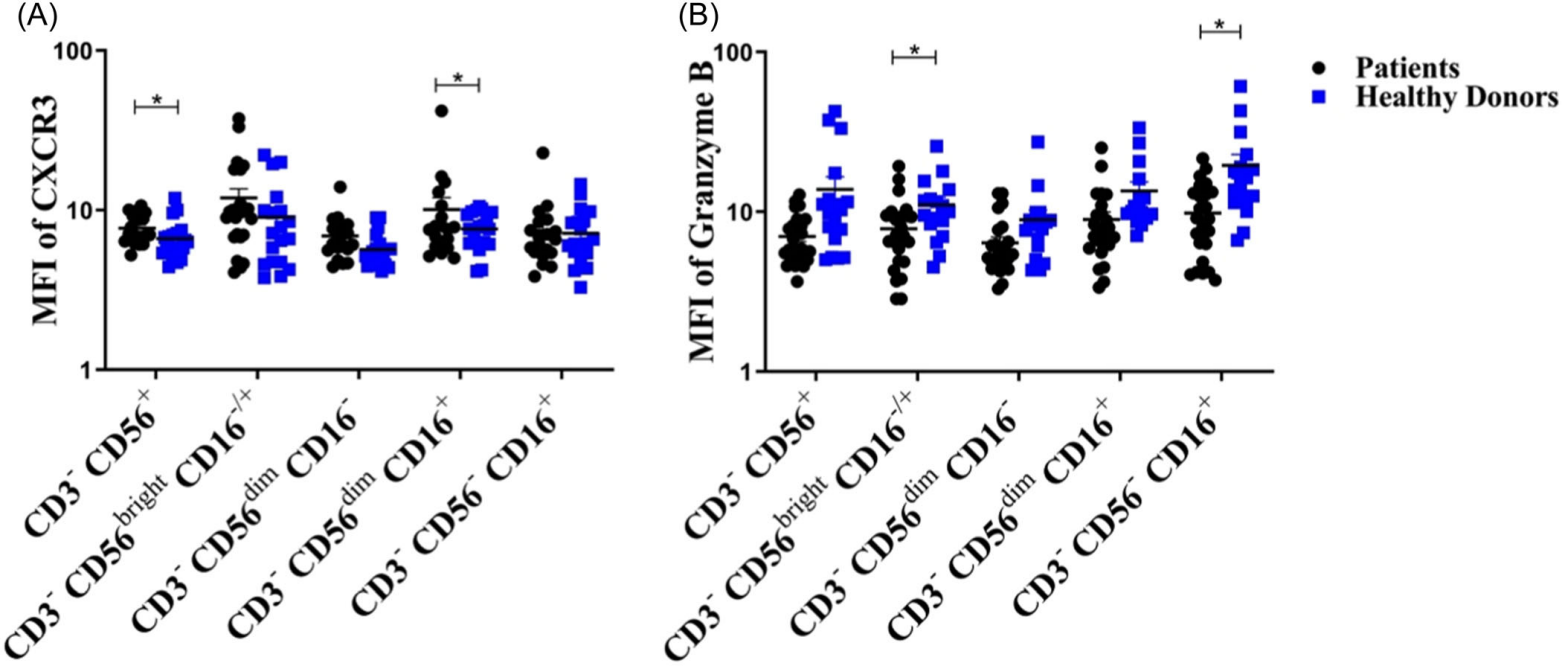
(A) Normalized mean fluorescent intensity (MFI) of CXCR3 on NK subsets (B) MFI of granzyme B in natural killer (NK) subsets in peripheral blood of patients with breast cancer and healthy donors. Data are analyzed by Mann–Whitney U test; *p‐value < .05.

We next determined and compared the mean percentages of functional molecules and receptors of NK cell subsets including NKG2D, NKP44, NKG2C, CD96 (evaluated on 20 out of 30 patient samples), CXCR3 (evaluated on 20 out of 30 patient samples), perforin, and granzyme B in patients with BC and HDs (Supporting Information S1: Table S1). Furthermore, the same data were compared between patients with different TNM stages (Supporting Information S1: Table S2) as well as LN involvement status (Supporting Information S1: Table S3). The statistical analyses revealed a significantly elevated mean (±SEM) expression of CXCR3 (based on MFI) in total NK cell population (7.72 ± 0.36 vs. 6.65 ± 0.42, *p* = .02) as well as conventional cytotoxic subset of NK cells (CD3^−^CD56^dim^CD16^+^) in the patients (6.90 ± 0.5 vs. 5.67 ± 0.32, *p* = .03, Figure 3A). In addition, the mean expression of granzyme B in conventional regulatory NK cells (CD3^−^CD56^bright^CD16^−^^/+^, 7.65 ± 0.67 vs. 11.12 ± 0.54, *p* = .03) and CD3^−^CD56^−^CD16^+^ NK cells (9.08 ± 0.52 vs. 19.65 ± 0.12, *p* = .004) were observed to be lower in the patients than the ones in HDs (Figure 3B).

**Figure 4:**
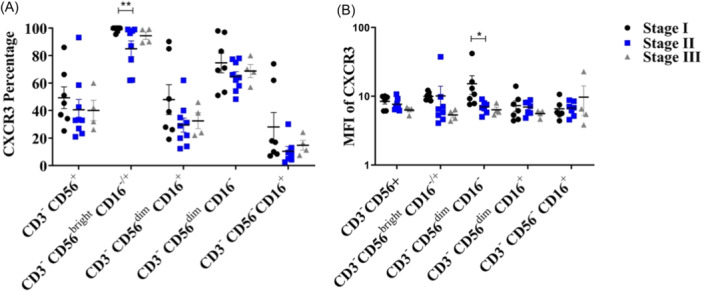
(A) Mean percentage of CXCR3 among natural killer (NK) cells and their subsets and (B) mean fluorescent intensity (MFI) of CXCR3 expression in NK subsets, in peripheral blood of the patients with breast cancer in different stages. Data are analyzed by Kruskal–Wallis *H* test, and Dunn's test was applied for *p*‐value adjustment in multiple comparisons; *p < .05, **p < .01.

The mean percentage of CXCR3^+^ conventional regulatory NK cells (CD3^−^CD56^bright^ CD16^−/+^) (99.58 ± 0.41 vs. 91.64 ± 3.26, *p* = .008) and mean expression level of CXCR3 in CD56^dim^CD16^−^ NK cells (*p* = .01) was revealed to be increased in the seven patients with stage I compared to nine patients with stage II. Moreover, a negative correlation was observed between these two NK cell subtypes and the stage of the disease (*R* = −0.557 with *p* = .02, and *R* = −0.661 with *p* = .004, respectively, Figure 4A,B).

The frequencies of granzyme B^+^ CD56^−^CD16^+^NK cells (64.46 ± 16.95 vs. 24.26 ± 8.82, *p* = .02) and CXCR3^+^CD56^dim^CD16^−^ (86.55 ± 7.04 vs. 63.21 ± 2.98, *p* = .02) was revealed to be significantly higher in the patients with grade I in comparison with those in grade II. In addition, a negative correlation was demonstrated between the mean expression of granzyme B in conventional cytotoxic NK cells and grade of the disease (*R* = −0.420, *p* = .04).

The mean expression of CXCR3 (based on MFI) in CD56^dim^CD16^−^ NK cells showed a higher level in the patients with tumor‐free lymph nodes (LN−) compared to the ones with tumor‐involved lymph nodes (LN+) (13.3 ± 3.73 vs. 6.48 ± 0.38, *p* = .02). Moreover, there was a significant negative correlation between mean expression of CXCR3 in CD56^dim^CD16^−^ NK cells and lymph node involvement (*R* = −0.487, *p* = .03). No further significant differences were observed in the frequency of NK cell subsets and their functional molecules between patients with BC and HDs.

## 4 DISCUSSION

NK cells are CD3^−^ innate lymphocytes with different subsets, which play pivotal roles in tumor immunity by secreting prepacked intracellular cytotoxic granules and/or applying surface effector molecules.^15^ In the current study, we investigated peripheral blood NK cell subsets and their functional molecules in patients with BC and HDs. The differences in the expression of NK cell functional molecules were then assessed between patients with different clinical and pathological characteristics including clinical stages, lymph node involvement, tumor size, and histological grade.

Our data showed that total human peripheral blood NK (CD3^−^CD56^+^) cells encompass about 15% of lymphocytes in the patients with BC and HDs (15.85 and 15.18, respectively) with no differences between the two groups. The finding is consistent with previous studies in which total NK cell percentages were reported to be 10%–15% of lymphocyte population in peripheral blood of healthy individuals.^12^, ^15^, ^16^ Considering the conventional classification of NK cells, our data indicated the regulatory subset (CD3^−^CD56^bright^CD16^−^^/+^) to be constituted 0.63% of lymphocytes in both groups, while cytotoxic NK cells (CD3^−^CD56^dim^CD16^+^) to be 9.88% and 12.44% in patients and HDs, respectively. Although these frequencies in our study did not pass statistical significance, Mamessier et al. have already reported an increase in the frequency of the regulatory subset as well as a decrease in cytotoxic subset in the peripheral blood of patients with BC referred to Institut PaoliCalmettes, France.^11^ Consistent with our results, a study in Netherlands showed that the percentages of these two subsets in the peripheral blood were not statistically different between the patients with colorectal cancer and healthy controls.^17^

Besides the aforementioned main conventional NK cell subsets, we observed two other NK subsets in the patients with BC and HDs; the exhausted or dysfunctional CD56^−^CD16^+^ NK cells (1.36% vs. 1.62%, respectively) and the immature CD56^dim^CD16^−^ NK cells (0.69% vs. 1.01%, respectively). Functionally, the exhausted and immature NK subsets have impaired cytotoxicity and decreased cytokine production capacity, and the existence of these unusual subsets was reported in different chronic inflammatory conditions such as HIV ^18^ and HCV infections,^19^ myasthenia gravis^20^ and melanoma.^21^ A significantly increased frequencies of the exhausted and immature NK subsets was reported in the peripheral blood of patients with advanced invasive BC, and was associated with tumor progression.^11^ The finding in advanced invasive BC is consistent with what previously reported in patients with HIV,^22^ suggesting a role for these two subsets in immune exhaustion. The suggestion, however, needs to be clarified by further studies.

Despite the applicability of the conventional classification of NK cells, from the last decade, the new classification of NK cell subsets was introduced based on the expression of CD11b (Mac‐1), a well‐known mature marker of NK cells, and CD27, a member of the tumor necrosis factor (TNF) family.^12^ Accordingly, we then investigated the distribution of NK cells based on this new classification. In view of that, the percentages of cytotoxic (CD3^−^CD56^+^CD11b^+^CD27^−^), regulatory (CD3^−^CD56^+^CD27^+^CD11b^+/^^−^) and tolerant NK cells (CD3^−^CD56^+^CD27^−^CD11b^−^) in lymphocyte population were observed to be respectively 11.26%, 0.94%, and 0.82% in the patients and 9.73%, 0.99%, and 0.48% in HDs. Statistical analyses indicated no significant differences between the two groups. These results are in line with our previous data on the percentages of different NK cell subsets infiltrated to TME (tumor‐infiltrating NK, TINK) in the patients with BC,^14^ Accordingly, we did not observe significant differences in frequencies of conventional and new NK cell subsets between patients with BC in various stages and lymph node involvement status.^14^ The increased percentages of CD27^−^ CD11b^−^ NK cells, as a dysfunctional (tolerant) NK cell subset, were previously shown to be associated with tumor progression in hepatocellular carcinoma and lung cancer.^23^, ^24^ Observing no differences between our cases and controls may come from the differences in sample size, tumor subtypes, and patient's ethnicity.

Besides evaluating the percentages of NK cell subsets based on conventional and new classification, we assessed the expression pattern of different functional molecules by NK cells. For such evaluation, we considered the conventional classification in which total NK cells are identified by CD3^−^CD56^+^ phenotype. Accordingly, we could not find any differences between cases and controls in terms of NK cell percentages positive for functional molecules NKG2D (88% vs. 84%, respectively), NKG2C (23% vs. 19%, respectively), NKp44 (12% vs. 9%, respectively), CD96, as a NK cell immune checkpoint (17% vs. 18%, respectively), and CXCR3 (44% vs. 34% respectively, *p* = .09). While we evaluated the expression of CXCR3 on 20 out of 30 patient samples (including seven patients with stage I, nine patients with stage II, and four patients with stage III) due to resource limitations, investigating the level of expression per cell (based on normalized mean fluorescent intensity, MFI) revealed that NK cells from patients with BC express CXCR3 significantly more than HDs. This significant finding was observed both when we considered total NK cells (7.72 vs. 6.65, respectively) as well as conventional cytotoxic NK cell subset (6.90 vs. 5.67). The expression of CXCR3 ligands (CXCL9/10/11) has been reported in cancer cells,^25^, ^26^ and breast carcinoma cell lines.^27^, ^28^ Studies indicated that tumors cells are able to secret CXCR3 ligand as a part of their immune evasion mechanisms; leading to recruitment of peripheral blood NK cells to TME.^29^ Therefore, higher expression of CXCR3 on NK cells in patients with BC might be associated with the potency of NK cell infiltration to tumor site. We also observed that the MFI of CXCR3 in immature (CD56^dim^CD16^−^) NK cells is significantly increased in the patients with stage I compared to the ones in stage II. A significant negative correlation was also observed between MFI of CXCR3 in this subset and lymph node involvement (*R* = −0.487). These two findings in immature NK cell subsets further imply that the recruitment of NK cells to TME, for example, via CXCL10‐CXCR3 axis,^30^ may not necessarily support antitumor immunity, as immature subset have been reported to have roles in cancer progression.^31^ The role of this subset in TME, however, remains to be elucidated.

Intracellular granzyme B was observed to be expressed in 47% of total NK cell population in patients, the figure to be 69% in HDs, revealing a borderline significance decrease in the expression of this cytotoxic molecules in patients' NK cells (*p* = .05). Investigating the level of expression per cell revealed NK cells from patient to express granzyme B significantly lower than HDs. This finding was observed as a nonsignificant trend when considering total NK cells but significant in case of conventional regulatory (7.65 vs. 11.12) as well as dysfunctional NK cells (9.08 vs. 19.65). Although we observed no significant differences, neither in the percentages nor in the mean expression of the other cytotoxic molecule; intracellular perforin, between patients and controls (92% vs. 91% and 21.21 vs. 22.84 for percentages and MFI, respectively), the findings collectively suggest less cytotoxic potency of the mentioned NK cell subsets in the patients with BC compared to HDs. Moreover, we observed a significant decrease in the percentage of granzyme B expressing CD56^−^CD16^+^ NK cells in the patients with grade II of BC compared to grade I (24.26 vs. 64.46). Our analyses also showed a negative correlation between the mean expression of granzyme B in conventional cytotoxic NK cells and the grade of the disease (R = −0.42), which collectively suggest the decrease in the level of granzyme B expression in patients by the progression of the tumor. Intracellular granzymes A, B, H, and K, as well as perforin, are the preformed weapons of NK cells required for target cell destruction and apoptosis.^32^, ^33^ In this regard, there is a report on significantly decreased percentage of perforin‐expressing NK cells (but not granzyme B) in different types of cancers.^34^ In addition, lower level of granzyme H was reported in the sera of Iranian patients with BC compared to HDs.^35^ Furthermore, studies showed that the low serum level of granzyme B is related to worse clinical outcomes and ineffective response to immune checkpoint (IC) blockade.^36^ Lower granzyme B, at least by some NK cell subsets, besides higher CXCR3 expression per total NK cells collectively suggest that NK cells in BC have a potency to be recruited to the tumor site, but these cells, or at least some of their subsets, may not be able to render their cytotoxic effects. This hypothesis however needs functional studies to be clarified.

## 5 CONCLUSION

The higher mean expression of CXCR3 on the total NK cells and its conventional cytotoxic subset in the peripheral blood of the patients with BC compared to HDs may be linked to chemotactic recruitment and migration of NK cells. However, further functional studies are recommended to evaluate the specific effects of this increased expression on NK cell biology and the outcome of breast tumors. Moreover, the lower mean expression of granzyme B in conventional regulatory as well as exhausted NK cells in the patients compared to the ones in HDs suggest less cytotoxic activity of these NK cell subsets in the patients with BC, supporting the accumulation of NK subsets with dysfunctional phenotype in the peripheral blood of patients with BC. Taken together, these findings suggest that breast tumors might render alteration in NK cell phenotype, subsets, and functional molecules in favor of its progression. Our observations could provide insights into NK cell behavior and may have implications for NK cell‐based immunotherapy in cancer.

## AUTHOR CONTRIBUTIONS

Reza Darvishvand and Somayeh Rezaeifard conceived and designed the experiments, performed the lab work, analyzed the data, and wrote the paper. Sedigheh Tahmasebi collected the samples and data. Razie Kiani designed the experiments, performed the lab work, and revised the paper. Nasrollah Erfani and Zahra Faghih conceived and designed the experiments, wrote and revised the paper.

## CONFLICT OF INTEREST STATEMENT

The authors declare no conflict of interest.

## Supporting information

Supporting information.

## Data Availability

The data that support the findings of this study are available from the corresponding author upon reasonable request.
