# Supplementary material for: Natural killer cell subsets and their functional molecules in peripheral blood of the patients with breast cancer
Source: Immun Inflamm Dis. 2024 Apr 23;12(4):e1255. doi: 10.1002/iid3.1255 (PMC11037257; doi:10.1002/iid3.1255)
Supplement: Supplementary file 1 — Supporting information. [file IID3-12-e1255-s001.docx]

**Supplementary data**

**Table S1**. The mean percentage of functional molecules and receptors of NK cell subsets in peripheral blood of breast cancer patients (P) and healthy donors (HDs)

| **Cell Subsets** | **CD Markers** | **Mean ± SEM**  **P** | **P Value ^a^** |
| --- | --- | --- | --- |
| **Applied Gate: Total NK cells** |  | **Mean ± SEM**  **HD** |  |
| NKG2D^+^ NK cells | NKG2D^+^CD3^-^CD56^+^ | 88.87 ± 2.52 | P=0.08 |
|  |  | 85.52 ± 1.35 |  |
|  | NKG2D^+^CD3^-^CD56^bright^CD16^-/+^ | 88.35 ± 2.26 | P=0.10 |
|  |  | 84.64 ± 2.31 |  |
|  | NKG2D^+^CD3^-^CD56^dim^CD16 ^-^ | 64.64 ± 3.30 | P=0.06 |
|  |  | 53.94 ± 4.20 |  |
|  | NKG2D^+^CD3^-^CD56^dim^CD16 ^+^ | 96.14 ± 0.67 | P=0.07 |
|  |  | 93.33 ± 1.37 |  |
|  | NKG2D^+^CD3^-^CD56^-^CD16^+^ | 37.16 ± 4.31 | P=0.09 |
|  |  | 23.25 ± 2.45 |  |
| *****CD96^+^ NK cells | CD96^+^CD3^-^CD56^+^ | 17.85 ± 2.48 | P=0.74 |
|  |  | 18.43 ± 2.58 |  |
|  | CD96^+^CD3^-^CD56^bright^CD16^-/+^ | 38.85 ± 5.21 | P=0.43 |
|  |  | 31.34 ± 5.30 |  |
|  | CD96^+^CD3^-^CD56^dim^CD16 ^-^ | 27.58 ± 2.92 | P=0.52 |
|  |  | 24.35 ± 2.88 |  |
|  | CD96^+^CD3^-^CD56^dim^CD16 ^+^ | 16.26 ± 2.50 | P=0.60 |
|  |  | 17.56 ± 2.69 |  |
|  | CD96^+^CD3^-^CD56^-^CD16^+^ | 12.70 ± 1.54 | P=0.06 |
|  |  | 17.28 ± 2.14 |  |
| *****CXCR3^+^ NK cells | CXCR3^+^CD3^-^CD56^+^ | 44.27 ± 4.50 | P=.09 |
|  |  | 34.93 ± 3.61 |  |
|  | CXCR3^+^CD3^-^CD56^bright^CD16^-/+^ | 95.36 ± 1.49 | P=0.10 |
|  |  | 83.64 ± 6.23 |  |
|  | CXCR3^+^CD3^-^CD56^dim^CD16 ^-^ | 68.01 ± 3.28 | P=0.30 |
|  |  | 62.32 ± 3.70 |  |
|  | CXCR3^+^CD3^-^CD56^dim^CD16 ^+^ | 37.33 ± 5.09 | P=0.51 |
|  |  | 31.75 ± 3.73 |  |
|  | CXCR3^+^CD3^-^CD56^-^CD16^+^ | 18.40 ± 4.35 | P=0.83 |
|  |  | 13.93 ± 1.97 |  |
| NKG2C^+^ NK cells | NKG2C^+^CD3^-^CD56^+^ | 23.82 ± 2.88 | P=0.42 |
|  |  | 19.53 ± 2.53 |  |
|  | NKG2C^+^CD3^-^CD56^bright^CD16^-/+^ | 37.77 ± 5.28 | P=0.57 |
|  |  | 32.68 ± 4.58 |  |
|  | NKG2C^+^CD3^-^CD56^dim^CD16 ^-^ | 16.53 ± 2.71 | P=0.79 |
|  |  | 14.60 ± 2.03 |  |
|  | NKG2C^+^CD3^-^CD56^dim^CD16 ^+^ | 22.59 ± 3.06 | P=0.63 |
|  |  | 19.80 ± 2.61 |  |
|  | NKG2C^+^CD3^-^CD56^-^CD16^+^ | 11.86 ± 1.99 | P=0.35 |
|  |  | 13.77 ± 1.81 |  |
| NKp44^+^ NK cells | NKp44^+^ CD3^-^CD56^+^ | 12.57 $\pm$ 1.47 | P=0.22 |
|  |  | 9.41 $\pm$ 0.97 |  |
|  | NKp44^+^CD3^-^CD56^bright^CD16^-/+^ | 48.43 $\pm$ 5.14 | P=0.33 |
|  |  | 38.39 $\pm$ 4.45 |  |
|  | NKp44^+^ CD3^-^CD56^dim^CD16 ^-^ | 12.78 $\pm$ 1.60 | P=0.24 |
|  |  | 7.68 $\pm$ 0.75 |  |
|  | NKp44^+^ CD3^-^CD56^dim^CD16 ^+^ | 11.94 $\pm$ 1.53 | P=0.34 |
|  |  | 8.91$\pm$ 1.02 |  |
|  | NKp44^+^ CD3^-^CD56^-^CD16 ^-^ | 5.28 $\pm$ 0.78 | P=0.86 |
|  |  | 5.60 $\pm$ 1.13 |  |
| Granzyme B ^+^ NK cells | Granzyme^+^CD3^-^CD56^+^ | 47.20 ± 6.67 | P=0.05 |
|  |  | 69.92 ± 3.76 |  |
|  | Granzyme^+^CD3^-^CD56^bright^CD16^-/+^ | 21.23 ± 7.03 | P=0.10 |
|  |  | 10.80 ± 3.38 |  |
|  | Granzyme^+^CD3^-^CD56^dim^CD16 ^-^ | 28.76 ± 6.57 | P=0.16 |
|  |  | 33.46 ± 5.21 |  |
|  | Granzyme^+^CD3^-^CD56^dim^CD16 ^+^ | 49.35 ± 6.83 | P=0.07 |
|  |  | 68.71 ± 5.08 |  |
|  | Granzyme^+^CD3^-^CD56^-^CD16^+^ | 29.03 ± 6.92 | P=0.09 |
|  |  | 28.35 ± 4.26 |  |
| Perforin^+^ NK cells | Perforin^+^CD3^-^CD56^+^ | 92.44 ± 1.61 | P=0.33 |
|  |  | 91.81 ± 1.04 |  |
|  | Perforin^+^CD3^-^CD56^bright^CD16^-/+^ | 51.52 ± 5.36 | P=0.09 |
|  |  | 29.67 ± 5.21 |  |
|  | Perforin^+^CD3^-^CD56^dim^CD16 ^-^ | 71.02 ± 3.05 | P=0.08 |
|  |  | 58.60 ± 3.16 |  |
|  | Perforin^+^CD3^-^CD56^dim^CD16^+^ | 97.46 ± 1.48 | P=0.17 |
|  |  | 97.43 ± 0.33 |  |
|  | Perforin^+^CD3^-^CD56^-^CD16^+^ | 46.22 ± 4.20 | P=0.30 |
|  |  | 50.47 ± 4.14 |  |

* CXCR3 and CD96 were evaluated on 20 out of 30 patient samples

^a^ Data are analyzed by Mann–Whitney U test

**Table S2**. The mean percentage of NK cell subsets, their functional molecules and receptors in peripheral blood of breast cancer patients with different stages

|  | | **Stage I** | **Stage II** | **Stage III** | **P value ^a^** |
| --- | --- | --- | --- | --- | --- |
| **Cell Subsets** | | **Mean ± SEM** | **Mean ± SEM** | **Mean ± SEM** |  |
| **Applied Gate** | **Lymphocytes** |  |  |  |  |
| CD3^-^CD56^+^ | | 11.13 ± 2.01 | 14.02 ± 1.78 | 11.79 ± 2.57 | P=0.61 |
| CD3^-^CD56^bright^CD16^-/+^ | | 0.78 ± 0.32 | 0.50 ± 0.08 | 0.54 ± 0.15 | P=0.72 |
| CD3^-^CD56^dim^CD16^-^ | | 0.72 ± 0.13 | 0.67 ± 0.15 | 0.64 ± 0.13 | P=0.63 |
| CD3^-^CD56^dim^CD16^+^ | | 10.92 ± 3.09 | 10.16 ± 1.75 | 6.55 ± 2.93 | P=0.32 |
| CD3^-^CD56^-^CD16^+^ | | 1.30 ± 0.32 | 1.19 ± 0.25 | 2.26 ± 0.59 | P=0.17 |
| CD3^-^CD56^+^CD27^+^CD11b^+/-^ | | 0.85 ± 0.14 | 0.93 ± 0.12 | 1.32 ± 0.20 | P=0.18 |
| CD3^-^CD56^+^CD27^-^CD11b^+^ | | 12.51 ± 2.64 | 11.18 ± 1.77 | 11.89 ± 1.48 | P=0.73 |
| CD3^-^CD56^+^CD27^-^CD11b^-^ | | 1.20 ± 0.30 | 0.74 ± 0.19 | 0.79 ± 0.06 | P=0.41 |
| CD3^+^CD56^+^ | | 7.5 ± 1.59 | 9.68 ± 1.8 | 4.22 ± 0.85 | P=0.09 |
| CD3^+^ cells | | 63.7 ± 5.57 | 66.06 ± 1.99 | 58.5 ± 6.13 | P=0.32 |
| Applied Gate | Total NK cells |  |  |  |  |
| NKG2D^+^CD3^-^CD56^+^ | percentage | 91.1 ± 2.55 | 89.70 ± 2.39 | 77.75 ± 14.98 | P=0.84 |
|  | MFI | 13.41 ± 1.73 | 11.34 ± 1.12 | 14.02 ± 2.64 | P=0.41 |
| NKG2D+ CD3^-^CD56^bright^CD16^-/+^ | percentage | 93.77 ± 3.08 | 85.41 ± 4.13 | 88.32 ± 4.55 | P=0.33 |
|  | MFI | 20.65 ± 3.98 | 25.82 ± 9.65 | 12.75 ± 2.83 | P=0.45 |
| NKG2D^+^CD3^-^CD56^dim^CD16^-^ | percentage | 64.70 ± 6.70 | 63.92 ± 4.93 | 59.7 ± 9.92 | P=0.88 |
|  | MFI | 19.39 ± 2.25 | 16.31 ± 1.65 | 16.18 ± 4.03 | P=0.37 |
| NKG2D^+^CD3^-^CD56^dim^CD16^+^ | percentage | 97.08 ± 0.89 | 95.64 ± 1.25 | 96.75 ± 1.46 | P=0.60 |
|  | MFI | 8.60 ± 1.11 | 8.26 ± 1.08 | 10.38 ± 2.52 | P=0.76 |
| NKG2D^+^CD3^-^CD56^-^CD16^+^ | percentage | 34.52 ± 8.37 | 35.9 ± 7.11 | 44.2 ± 12.26 | P=0.85 |
|  | MFI | 11.47 ± 2.07 | 9.83 ± 1.54 | 13.69 ± 4.22 | P=0.66 |
| *****CD96^+^CD3^-^CD56^+^ | percentage | 22.71 ± 6.67 | 13.78 ± 2.86 | 18.43 ± 5.66 | P=0.21 |
|  | MFI | 5.41 ± 0.59 | 5.18 ± 0.48 | 5.07 ± 0.46 | P=0.91 |
| *****CD96^+^CD3^-^CD56^bright^CD16^-/+^ | percentage | 46.44 ± 9.56 | 33.22 ± 9.02 | 39.84 ± 10.99 | P=0.43 |
|  | MFI | 7.90 ± 1.66 | 6.38 ± 0.59 | 6.05 ± 0.83 | P=0.84 |
| *****CD96^+^CD3^-^CD56^dim^CD16^-^ | percentage | 29.85 ± 6.45 | 26.01 ± 4.53 | 23.65 ± 5.04 | P=0.88 |
|  | MFI | 4.98 ± 0.48 | 4.64 ± 0.29 | 4.75 ± 0.44 | P=0.97 |
| *****CD96^+^CD3^-^CD56^dim^CD16^+^ | percentage | 22.06 ± 7.37 | 11.98 ± 2.59 | 16.02 ± 6.09 | P=0.34 |
|  | MFI | 5.00 ± 0.59 | 5.02 ± 0.44 | 4.92 ± 0.42 | P=0.85 |
| *****CD96^+^CD3^-^CD56^-^CD16^+^ | percentage | 12.57 ± 3.68 | 11.94 ± 1.86 | 12.7 ± 7.87 | P=0.74 |
|  | MFI | 4.35 ± 0.32 | 4.73 ± 0.2 | 4.82 ± 0.23 | P=0.40 |
| *****CXCR3^+^CD3^-^CD56^+^ | percentage | 53.31 ± 8.03 | 41.51 ± 8.51 | 40.07 ± 7.39 | P=0.20 |
|  | MFI | 8.43 ± 0.75 | 7.65 ± 0.54 | 6.28 ± 0.36 | P=0.25 |
| *****CXCR3^+^CD3^-^CD56^bright^ CD16^+^ | percentage | 99.58 ± 0.41 | 91.64 ± 3.26 | 94.47 ± 2.70 | P= 0.008  Stage I vs. II |
|  | MFI | 10.51 ± 1.65 | 11.49 ± 5.27 | 5.36 ± 0.47 | P=0.08 |
| *****CXCR3^+^CD3^-^CD56^dim^CD16^-^ | percentage | 75.33 ± 8.47 | 61.44 ± 3.51 | 68.8 ± 4.71 | P=0.30 |
|  | MFI | 16.22 ± 5.31 | 6.98 ± 0.51 | 6.35 ± 0.56 | P=0.01  Stage I vs II |
| *****CXCR3^+^CD3^-^CD56^dim^CD16^+^ | percentage | 52.86 ± 11.5 | 28.81 ± 6.62 | 32.62 ± 5.75 | P=0.10 |
|  | MFI | 7.64 ± 1.45 | 6.95 ± 0.56 | 5.6 ± 0.32 | P=0.40 |
| *****CXCR3^+^CD3^-^CD56^-^CD16^+^ | percentage | 31.59 ± 11.71 | 10.43 ± 3.55 | 14.87 ± 3.61 | P=0.10 |
|  | MFI | 6.57 ± 0.91 | 6.71 ± 0.58 | 9.69 ± 4.43 | P=0.80 |
| NKG2C^+^CD3^-^CD56^+^ | percentage | 24.87 ± 5.05 | 20.33 ± 4.31 | 27.7 ± 6.71 | P=0.48 |
|  | MFI | 6.19 ± 0.58 | 7.24 ± 0.68 | 6.16 ± 1.32 | P=0.57 |
| NKG2C^+^CD3^-^CD56^bright^CD16^-/+^ | percentage | 43.48 ± 9.87 | 32.85 ± 7.18 | 34.22 ± 17.7 | P=0.79 |
|  | MFI | 5.92 ± 0.51 | 8.55 ± 1.8 | 7.87 ± 2.12 | P=0.64 |
| NKG2C^+^CD3^-^CD56^dim^CD16^-^ | percentage | 15.54 ± 4.01 | 13.73 ± 3.09 | 13.71 ± 6.47 | P=0.66 |
|  | MFI | 5.75 ± 0.36 | 6.94 ± 0.92 | 7.14 ± 1.02 | P=0.44 |
| NKG2C^+^CD3^-^CD56^dim^CD16^+^ | percentage | 23.98 ± 5.49 | 20.38 ± 4.43 | 18.72 ± 6.87 | P=0.75 |
|  | MFI | 6.38 ± 0.73 | 7.44 ± 0.92 | 6.42± 1.17 | P=0.82 |
| NKG2C^+^CD3^-^CD56^-^CD16^+^ | percentage | 14.46 ± 5.07 | 9.16 ± 2.02 | 17.35 ± 7.21 | P=0.66 |
|  | MFI | 9.08 ± 2.72 | 6.20 ± 0.61 | 6.37 ± 1.33 | P=0.81 |
| NKp44^+^CD3^-^CD56^+^ | percentage | 14.55 ± 2.80 | 12.16 ± 2.07 | 12.08 ± 4.09 | P=0.62 |
|  | MFI | 5.69 ± 0.38 | 5.22 ± 0.25 | 7.65 ± 2.38 | P=0.22 |
| NKp44^+^CD3^-^CD56^bright^CD16^-/+^ | percentage | 51.75 ± 8.50 | 49 ± 7.59 | 45.64 ± 19.74 | P=0.81 |
|  | MFI | 7.03 ± 0.74 | 5.79 ± 0.48 | 6.79 ± 1.37 | P=0.30 |
| NKp44^+^CD3^-^CD56^dim^CD16^-^ | percentage | 14.15 ± 3.48 | 11.53 ± 1.77 | 15.98 ± 6.70 | P=0.93 |
|  | MFI | 5.41 ± 0.27 | 4.76 ± 0.17 | 6.01 ± 0.78 | P=0.06 |
| NKp44^+^CD3^-^CD56^dim^CD16^+^ | percentage | 13.17 ± 2.72 | 11.81 ± 2.41 | 11.38 ± 4.43 | P=0.88 |
|  | MFI | 5.27 ± 0.45 | 4.95 ± 0.31 | 5.47 ± 0.76 | P=0.60 |
| NKp44^+^CD3^-^CD56^-^CD16^+^ | percentage | 7.16 ± 1.95 | 4.06 ± 0.66 | 4.94 ± 2.59 | P=0.54 |
|  | MFI | 4.85 ± 0.28 | 6.48 ± 0.83 | 5.83 ± 1.36 | P=0.36 |
| Granzyme^+^CD3^-^CD56^+^ | percentage | 5.25 ± 15.11 | 50.64 ± 10.03 | 33.5 ± 14.48 | P=0.78 |
|  | MFI | 7.99 ± 1.18 | 7.17 ± 0.64 | 5.7 ± 0.59 | P=0.64 |
| Granzyme^+^CD3^-^CD56^bright^CD16^-/+^ | percentage | 24.35 ± 14.69 | 27.35 ± 11.19 | 0.99 ± 0.49 | P=0.53 |
|  | MFI | 8.40 ± 1.81 | 4.69 ± 0.53 | 16 ± 00 | P=0.43 |
| Granzyme^+^CD3^-^CD56^dim^CD16^-^ | percentage | 27.65 ± 13.69 | 34.5 ± 10.45 | 12.52 ± 6.43 | P=0.82 |
|  | MFI | 8.2 ± 0.69 | 8.83 ± 1.82 | 12.19 ± 3.61 | P=0.08 |
| Granzyme^+^CD3^-^CD56^dim^CD16^+^ | percentage | 52.81 ± 15.19 | 50.37 ± 9.71 | 39.16 ± 22.08 | P=0.63 |
|  | MFI | 7.46 ± 1.32 | 6.35 ± 0.76 | 5.36 ± 0.28 | P=0.33 |
| Granzyme^+^CD3^-^CD56^-^CD16^+^ | percentage | 36.01 ± 15.16 | 30.79 ± 10.48 | 15.36 ± 9.32 | P=0.80 |
|  | MFI | 9.44± 1.40 | 9.67 ± 1.46 | 14.13 ± 3.59 | P=0.72 |
| Perforin^+^CD3^-^CD56^+^ | percentage | 94.80 ± 1.40 | 91.68 ± 2.94 | 92 ± 1.22 | P=0.42 |
|  | MFI | 38.73 ± 8.05 | 37.93 ± 6.51 | 46.2 ± 12.1 | P=0.61 |
| Perforin^+^CD3^-^CD56^bright^CD16^-/+^ | percentage | 61.60 ± 11.93 | 52.88 ± 7.02 | 32.57 ± 9.46 | P=0.31 |
|  | MFI | 9.66 ± 3.6 | 8.49 ± 1.48 | 13.62 ± 5.13 | P=0.25 |
| Perforin^+^CD3^-^CD56^dim^CD16^-^ | percentage | 76.24 ± 4.15 | 67.69 ± 4.64 | 71.42 ± 9.63 | P=0.74 |
|  | MFI | 15.37 ± 3.29 | 20.28 ± 4.38 | 16.57 ± 2.20 | P=0.83 |
| Perforin^+^CD3^-^CD56^dim^CD16^+^ | percentage | 98.82 ± 0.44 | 96.32 ± 2.76 | 98.57 ± 0.82 | P=0.97 |
|  | MFI | 38.17 ± 11.17 | 36.08 ± 9.88 | 28.75 ± 10.57 | P=0.84 |
| Perforin^+^CD3^-^CD56^-^CD16^+^ | percentage | 54.50 ± 8.78 | 46.87 ± 5.58 | 38.5 ± 12 | P=0.46 |
|  | MFI | 24.57 ± 4.75 | 28.01 ± 4.27 | 22.85 ± 11.56 | P=0.62 |

* CXCR3 and CD96 were evaluated on 20 out of 30 patient samples

^a^ Data are analyzed by Kruskal-Wallis H test, and Dunn’s test was applied for P value adjustment in multiple comparisons

**Table S3.** Mean percentage of NK cell subsets, their functional molecules and receptors in peripheral blood of breast cancer patients with different lymph node involvements

|  | | **LN-**  **Mean ± SEM** | **LN+**  **Mean ± SEM** | **P Value ^a^** |
| --- | --- | --- | --- | --- |
| **Cell Subsets** | |  |  |  |
| **Applied Gate** | **Lymphocytes** |  |  |  |
| CD3^-^CD56^+^ | | 12.09 ± 1.63 | 13.43 ± 1.77 | P=0.77 |
| CD3^-^CD56^bright^CD16^-/+^ | | 0.72 ± 0.22 | 0.48 ± 0.07 | P=0.73 |
| CD3^-^CD56^dim^CD16^-^ | | 0.88 ± 0.18 | 0.53 ± 0.07 | P=0.09 |
| CD3^-^CD56^dim^CD16^+^ | | 10.18 ± 2.08 | 9.59 ± 1.90 | P=0.34 |
| CD3^-^CD56^-^CD16^+^ | | 1.22 ± 0.22 | 1.51 ± 0.3 | P=0.87 |
| CD3^-^CD56^+^CD27^+^CD11b^+/-^ | | 0.85 ± 0.12 | 1.06 ± 0.12 | P=0.28 |
| CD3^-^CD56^+^CD27^-^CD11b^+^ | | 11.08 ± 1.93 | 12.12 ± 1.62 | P=0.64 |
| CD3^-^CD56^+^CD27^-^CD11b^-^ | | 0.98 ± 0.22 | 0.8 ± 0.19 | P=0.76 |
| CD3^+^CD56^+^ | | 7.81 ± 1.11 | 8.58 ± 1.90 | P=0.22 |
| CD3^+^ cells | | 65.35 ± 3.80 | 63.35 ± 2.45 | P=0.72 |
| Applied Gate | Total NK cells |  |  |  |
| NKG2D^+^CD3^-^CD56^+^ | percentage | 88.48 ± 2.52 | 87.86 ± 5.15 | P=0.56 |
|  | MFI | 12.44 ± 1.43 | 12.51 ± 1.16 | P=0.94 |
| NKG2D^+^CD3^-^CD56^bright^CD16^-/+^ | percentage | 90.58 ± 3.25 | 87.06 ± 3.6 | P=0.44 |
|  | MFI | 18.07 ± 3.07 | 25.46 ± 9.6 | P=0.97 |
| NKG2D^+^CD3^-^CD56^dim^CD16^-^ | percentage | 62.85 ± 5.10 | 64.13 ± 5.28 | P=0.13 |
|  | MFI | 18.95 ± 1.62 | 15.55 ± 2.01 | P=0.12 |
| NKG2D^+^CD3^-^CD56^dim^CD16^+^ | percentage | 95.67 ± 1.04 | 97.2 ± 0.86 | P=0.43 |
|  | MFI | 7.90 ± 0.98 | 9.74 ± 1.17 | P=0.28 |
| NKG2D^+^CD3^-^CD56^-^CD16^+^ | percentage | 29.35 ± 5.97 | 46.01 ± 6.92 | P=0.08 |
|  | MFI | 11.49 ± 1.49 | 10.70 ± 2.14 | P=0.53 |
| *****CD96^+^CD3^-^CD56^+^ | percentage | 19.38 ± 4.82 | 15.57 ± 2.99 | P=0.53 |
|  | MFI | 5.66 ± 0.49 | 4.85 ± 0.37 | P=0.51 |
| *****CD96^+^CD3^-^CD56^bright^CD16^-/+^ | percentage | 40.18 ± 8.26 | 36.96 ± 8.42 | P=0.65 |
|  | MFI | 7.74 ± 1.12 | 5.96 ± 0.53 | P=0.21 |
| *****CD96^+^CD3^-^CD56^dim^CD16^-^ | percentage | 25.92 ± 5.08 | 27.73 ± 4.03 | P=0.63 |
|  | MFI | 5.09 ± 0.35 | 4.47 ± 0.26 | P=0.34 |
| *****CD96^+^CD3^-^CD56^dim^CD16^+^ | percentage | 18.5 ± 5.24 | 13.42 ± 2.82 | P=0.58 |
|  | MFI | 5.30 ± 0.49 | 4.72 ± 0.34 | P=0.65 |
| *****CD96^+^CD3^-^CD56^-^CD16^+^ | percentage | 12.14 ± 2.37 | 12.32 ± 2.56 | P=0.64 |
|  | MFI | 4.56 ± 0.24 | 4.67 ± 0.22 | P=0.76 |
| *****CXCR3^+^CD3^-^CD56^+^ | percentage | 43.47 ± 7.16 | 46.77 ± 6.98 | P=0.71 |
|  | MFI | 8.51 ± 0.62 | 6.70 ± 0.23 | P=0.11 |
| *****CXCR3^+^CD3^-^CD56^bright^CD16^-/+^ | percentage | 95.11 ± 2.77 | 95.11 ± 1.85 | P=0.32 |
|  | MFI | 7.77 ± 1.77 | 10.06 ± 3.97 | P=0.96 |
| *****CXCR3^+^CD3^-^CD56^dim^CD16^-^ | percentage | 70.68 ± 6.03 | 65.13 ± 3.81 | P=0.78 |
|  | MFI | 13.3 ± 3.73 | 6.48 ± 0.38 | P=0.02 |
| *****CXCR3^+^CD3^-^CD56^dim^CD16^+^ | percentage | 40.4 ± 9.71 | 35.72 ± 4.91 | P=0.83 |
|  | MFI | 7.61 ± 0.98 | 6.05 ± 0.40 | P=.032 |
| *****CXCR3^+^CD3^-^CD56^-^CD16^+^ | percentage | 23.15 ± 8.67 | 14.20 ± 3.23 | P=0.81 |
|  | MFI | 6.54 ± 0.64 | 8.16 ± 2.16 | P=0.91 |
| NKG2C^+^CD3^-^CD56^+^ | percentage | 21.19 ± 3.88 | 24.95 ± 4.4 | P=0.71 |
|  | MFI | 25.64 ± 3.77 | 7.13 ± 0.77 | P=0.63 |
| NKG2C^+^CD3^-^CD56^bright^CD16^-/+^ | percentage | 38.8 ± 7.66 | 34.44 ± 8.14 | P=0.51 |
|  | MFI | 6.29 ± 0.44 | 8.90 ± 1.88 | P=0.63 |
| NKG2C^+^CD3^-^CD56^dim^CD16^-^ | percentage | 14.36 ± 3.24 | 14.30 ± 3.13 | P=0.97 |
|  | MFI | 6.20 ± 0.41 | 6.96 ± 0.93 | P=0.64 |
| NKG2C^+^CD3^-^CD56^dim^CD16^+^ | percentage | 20.48 ± 4.07 | 22.13 ± 4.55 | P=0.96 |
|  | MFI | 6.33 ± 0.51 | 7.50 ± 0.96 | P=0.68 |
| NKG2C^+^CD3^-^CD56^-^CD16^+^ | percentage | 12.25 ± 3.4 | 12.15 ± 3.04 | P=0.92 |
|  | MFI | 7.93 ± 1.77 | 6.34 ± 0.68 | P=0.61 |
| NKp44^+^CD3^-^CD56^+^ | percentage | 12.73 ± 1.87 | 12.40 ± 2.37 | P=0.88 |
|  | MFI | 5.74 ± 0.26 | 5.88 ± 0.72 | P=0.33 |
| NKp44^+^CD3^-^CD56^bright^CD16^-/+^ | percentage | 45.94 ± 6.17 | 51.10 ± 8.55 | P=0.61 |
|  | MFI | 6.74 ± 0.46 | 5.94 ± 0. 60 | P=0.12 |
| NKp44^+^CD3^-^CD56^dim^CD16^-^ | percentage | 12.18 ± 2.24 | 13.43 ± 2.38 | P=0.72 |
|  | MFI | 5.47 ± 0.26 | 5.04 ± 0.33 | P=0.10 |
| NKp44^+^CD3^-^CD56^dim^CD16^+^ | percentage | 11.56 ± 1.78 | 12.34 ± 2.60 | P=0.80 |
|  | MFI | 5.38 ± 0.28 | 4.96 ± 0.35 | P=0.20 |
| NKp44^+^CD3^-^CD56^-^CD16^+^ | percentage | 6.48 ± 1.26 | 4 ± 0.91 | P=0.06 |
|  | MFI | 5.24 ± 0.35 | 6.29 ± 0.87 | P=0.50 |
| Granzyme^+^CD3^-^CD56^+^ | percentage | 44.95 ± 10.14 | 49.93 ± 10.53 | P=0.60 |
|  | MFI | 6.91 ± 0.87 | 7.38 ± 0.59 | P=0.30 |
| Granzyme^+^CD3^-^CD56^bright^CD16^-/+^ | percentage | 19.20 ± 11.72 | 25.12 ± 10.45 | P=0.94 |
|  | MFI | 7.54 ± 1.38 | 6.1 ± 1.73 | P=0.86 |
| Granzyme^+^CD3^-^CD56^dim^CD16^-^ | percentage | 25.35 ± 9.84 | 32.47 ± 10.57 | P=0.90 |
|  | MFI | 8.17 ± 0.81 | 9.85 ± 1.93 | P=0.66 |
| Granzyme^+^CD3^-^CD56^dim^CD16^+^ | percentage | 47.7 ± 10.22 | 51.44 ± 11.02 | P=0.65 |
|  | MFI | 6.54 ± 0.91 | 6.57 ± 0.79 | P=0.79 |
| Granzyme^+^CD3^-^CD56^-^CD16^+^ | percentage | 25.43 ± 10.37 | 34.88 ± 10.96 | P=0.60 |
|  | MFI | 9.31 ± 1.05 | 10.98 ± 1.76 | P=0.80 |
| Perforin^+^CD3^-^CD56^+^ | percentage | 94.4 ± 1.05 | 91.22 ± 2.91 | P=0.51 |
|  | MFI | 33.23 ± 6.31 | 44.05 ± 6.22 | P=0.20 |
| Perforin^+^CD3^-^CD56^bright^CD16^-/+^ | percentage | 55.10 ± 9.21 | 49.44 ± 6.89 | P=0.73 |
|  | MFI | 10.83 ± 2.61 | 8.81 ± 1.77 | P=0.91 |
| Perforin^+^CD3^-^CD56^dim^CD16^-^ | percentage | 73.9 ± 3.89 | 68.12 ± 4.79 | P=0.70 |
|  | MFI | 14.63 ± 2.60 | 21.15 ± 4.17 | P=0.30 |
| Perforin^+^CD3^-^CD56^dim^CD16^+^ | percentage | 98.89 ± 0.36 | 96.21 ± 2.76 | P=0.90 |
|  | MFI | 33.41 ± 7.57 | 37.11 ± 10.00 | P=0.92 |
| Perforin^+^CD3^-^CD56^-^CD16^+^ | percentage | 49.3 ± 6.69 | 46.42 ± 5.85 | P=0.74 |
|  | MFI | 24.82 ± 5.8 | 27.37 ± 3.62 | P=0.57 |

* CXCR3 and CD96 were evaluated on 20 out of 30 patient samples

^a^ Data are analyzed by Mann–Whitney U test
